# Mandibles and labrum-epipharynx of tiger beetles: basic structure and evolution (Coleoptera, Carabidae, Cicindelitae)

**DOI:** 10.3897/zookeys.147.2052

**Published:** 2011-11-16

**Authors:** George E. Ball, John H. Acorn, Danny Shpeley

**Affiliations:** 1 Department of Biological Sciences, University of Alberta, Edmonton, Alberta, Canada T6G 2E9; 2 Department of Renewable Resources, University of Alberta, Edmonton, Alberta, Canada T6G 2H1

**Keywords:** comparative morphology, mouthparts, pre-oral mill, evolution, Coleoptera, Trachypachidae, Carabidae, Carabitae, Nebriitae, Cicindelitae

## Abstract

Using for comparison with, and as outgroups for, supertribe Cicindelitae, we describe and illustrate the mandibles and labrum-epipharynx of the basal geadephagans *Trachypachus gibbsii* LeConte, 1861 (family Trachypachidae), and family Carabidae: *Pelophila rudis* (LeConte, 1863) (supertribe Nebriitae, tribe Pelophilini) and *Ceroglossus chilensis* (Eschscholtz, 1829) (supertribe Carabitae, tribe Ceroglossini). The range and pattern of variation in structure of mandibles and labrum-epipharynx within the supertribe Cicindelitae was assessed using scanning-electron (SEM) images of these structures in nine exemplar taxa: *Amblycheila baroni* (Rivers, 1890), *Omus californicus* (Eschscholtz, 1829) and *Picnochile fallaciosa* (Chevrolat, 1854) (representing the Amblycheilini); *Manticora tuberculata* (DeGeer, 1778) (representing the Manticorini): *Tetracha carolina* (Linnaeus, 1767) (representing the Megacephalini); *Pogonostoma chalybeum* (Klug, 1835) (representing the Collyridini); and *Therates basalis* Dejean, 1826, *Oxycheila* species, and *Cicindela longilabris* Say, 1824 (representing the Cicindelini). An evolutionary transformation series was postulated for the mandibles and labrum-epipharynx, based on a reconstructed phylogenetic sequence, which, in turn, was based on morphological and DNAevidence.Principal features of the transformation series for the mandibles included development of a densely setose basal face; wide quadridentate retinaculum; a lengthened incisor tooth; a multidentate terebra (one to five teeth; two-three most frequent), followed by subsequent loss of one or more such teeth; development of a diastema in the occlusal surface; development and subsequent loss of scrobal setae, and reduction and loss of the scrobe. Principal features of the transformation series for the labrum included evolution of form from transverse, sub-rectangular to elongate almost square, to triangular; position and number of setae evolved from dorsal to insertion on the apical margin, the number increased from 8-10 to as many as 36, and decreased to as few as four. The epipharynx broadened evolutionarily, the pedium evolving in form from narrow, triangular and nearly flat, to broad, palatiform, and markedly convex; anterior parapedial setae both increased and decreased in number, and in orientation, from a row parallel to the parapedial ridge to a setal row extended forward at about a right angle to the latter.

## Introduction

In their review of variation in mandibular structure within the coleopteran suborder Adephaga, Acorn and Ball (1991) unfortunately did not include the distinctive but complex (and therefore difficult to interpret) mandibles of the tiger beetles (Carabidae: Cicindelitae). Since then, Ball et al (1995: 302-311) provided the basis for inclusion of the epipharynx in systematic studies, but did not include the tiger beetles or other basal geadephagan lineages. We (GEB, JHA) decided to fill in these gaps in knowledge of geadephagan structure, and to do so, enlisted in the cause our willing and able colleague, Danny Shpeley.

Our initial investigation of tiger beetle mandibles revealed for the Geadephaga a combination of unique features and in them, substantial variation. To understand these aspects, we sought an orienting principle in the relatively recent studies and postulates of tiger beetle evolution, admirably summarized by Pearson and Vogler (2001: 43-51). We turned to analyses (Maddison et al 1999) of related basal stocks of geadephagans to seek the antecedents of the tiger beetle mandibles and labrum-epipharynx. Tiger beetles have been placed as a supertribe (Cicindelitae) within the Carabidae (Erwin 1985: 467, Erwin 2007: 171, Erwin and Pearson 2008) or as a separate family, the Cicindelidae (Cassola 2001, Pearson and Vogler 2001, Deuve 2004:31), or as tribe Cicindelini (Liebherr and Will 1998: 151). Recent phylogenetic analyses of the Geadephaga have consistently placed tiger beetles with the Carabidae, in some instances giving this group a basal position within the carabid phylogeny (Erwin and Pearson 2008, Deuve 1994, Maddison et al. 1999: 104, Figure 1 (one alternative placement)) and in others placing the group higher in the tree (Arndt and Putchkov 1997, Beutel and Haas 1996: 201, Figure 1; Liebherr and Will 1998: 142, Figure 57A; Maddison et al. 1999 (another alternative placement)). Maddison et al. (1999: 115, Figure 6) place Trachypachidae as the adelphotaxon for the remaining Geadephaga as do Dressler and Beutel (2010).

In this paper, we illustrate and describe the mandibles and labrum-epipharynx of tiger beetles representing the five tribes here recognized (Amblycheilini, Manticorini, Megacephalini, Collyridini, and Cicindelini). We arrange them in a phylogenetic sequence, based on a postulated evolutionary pattern (Pearson and Vogler 2001: 46), and relate this series to the form of the mouthparts in Trachypachidae, Nebriitae, and Carabitae, the latter three taxa representing the three mouthpart configurations that may have preceded that of the tiger beetles in an evolutionary sense.

We are pleased to dedicate this study in comparative morphology to Ross and Joyce Bell, Department of Biology, University of Vermont, Burlington, Vermont, in recognition of their contributions to the study of tiger beetles, the use of mandibles as character systems, and more generally to the field of adephagan systematics (e.g., Bell 1966). Especially appropriate to note in the context of the present contribution are: Ross’ treatment of the North American Chlaeniini (Bell 1960), in which mandibular form and size were shown to be a useful diagnostic feature in classification; and his study of the mouthparts of rhysodine carabids (Bell 1994), whose mandibles he showed to function as a sheath for the underlying maxillae, and to be non-biting. His interest in tiger beetles was demonstrated through co-authoring a field guide to cicindelids (Leonard and Bell 1991).

## Material and methods

### Material

We examined 12 specimens with SEM, and an additional 37 with light (Wild M5 and M3 stereoscope) microscopy, (Table 1, Appendix). These specimens are housed in the E. H. Strickland Entomological Museum, University of Alberta (UASM), Royal Alberta Museum, Edmonton, Alberta (RAMC), California Academy of Science, San Francisco, California (CASC), John H. Acorn Collection, Edmonton, Alberta (JHAC), and Ronald L. Huber Collection, Bloomington, Minnesota (RLHI).

**Table 1.** Names, sex, and classification of exemplar individuals and species with SEM-illustrated mandibles and labrum-epipharynx.

Family TRACHYPACHIDAE
Tribe TRACHYPACHIDINI
*Trachypachus* Motschulsky
***Trachypachus gibbsii* LeConte, 1861** (male)
Family CARABIDAE
Supertribe CARABITAE
Tribe CEROGLOSSINI
*Ceroglossus* Solier
***Ceroglossus chilensis* (Eschscholtz, 1829)** (male)
Supertribe CICINDELITAE
Tribe AMBLYCHEILINI
*Amblycheila* Say
***Amblycheila baroni* (Rivers, 1890)** (female)
*Omus* Dejean
***Omus californicus* (Eschscholtz, 1829)** (male)
*Picnochile* Motschulsky
***Picnochile fallaciosa* (Chevrolat, 1835**) (female)
Tribe MANTICORINI Fabricius
*Manticora* Fabricius
***Manticora tuberculata* (DeGeer, 1778)** (female)
Tribe MEGACEPHALINI
*Tetracha* Hope
***Tetracha carolina* (Linnaeus, 1767)** (male)
Tribe COLLYRIDINI
*Pogonostoma* Klug
***Pogonostoma chalybeum* (Klug, 1835)** (male)
Tribe CICINDELINI
*Therates* Latreille
***Therates basalis* Dejean, 1826**(female)
*Oxycheila* Dejean
***Oxycheila species***, male
*Cicindela* Linnaeus
***Cicindela longilabris* Say, 1824** (male)
Supertribe NEBRIITAE
Tribe PELOPHILINI
*Pelophila* Dejean
***Pelophila rudis* (LeConte, 1863)**(female)

### Methods

**Taxon and specimen selection.** For the cicindelites, we chose exemplar specimens to represent the five currently recognized tribes: Amblycheilini, Manticorini, Megacephalini, Collyridini and Cicindelini. For comparative purposes, we chose representatives from basal geadephagan lineages: Trachypachidae (*Trachypachus gibbsii* (LeConte, 1861)), the putative adelphotaxon of the caraboid stock; and Carabidae— Carabitae-Ceroglossini (*Ceroglossus chilensis* (Eschscholtz, 1829)), and Nebriitae-Pelophilini (*Pelophila rudis* (LeConte, 1863)). These groups of Carabidae represent different feeding types (carabites, like cicindelites, primarily predatory fluid feeders; and nebriites, particulate feeders; see Evans and Forsythe (1985: 115).

**Specimen preparation.** Standard techniques were used to prepare specimens for examination with the SEM. Each specimen was relaxed and cleaned in warm water and the sclerites of interest were removed from the head capsule and sputter coated with gold before SEM images were prepared. For light microscopy, specimens were relaxed, cleaned, and the mandibles spread to their fullest extent. For some but not all taxa, the labrum epipharynx was excised and point-mounted.

**SEM image preparation.** Three aspects for both left and right mandibles are shown: dorsal, occlusal, and ventral; lateral aspects were noted, but not illustrated. For the labrum-epipharynx, the dorsal surface is the labrum, while the ventral surface is the epipharynx, keeping in mind that the epipharynx can also be thought of as the dorsal surface of the preoral cavity.

**Photographs.** The mouthparts of 12 taxa examined are also illustrated *in situ*, with the mandibles widely spread, hopefully providing better perspective on the relative coverage of the adducted mandibles by the labrum-epipharynx for each taxon, as well as some sense of potential occlusal relationships among mandible features, and bilateral asymmetry. Photographs were taken hand-held with a Nikon D300s camera set at ISO 800 and 1/200th of a second, equipped with an AF-S Micro-Nikkor 105mm lens set at f32, a TC-17EII (1.7X) teleconverter, and the Nikon R1C1macro flash system with two flash heads, each equipped with hand made, double-layered, frosted Mylar light diffusers. These images were presented on two color plates.

**Identification of structural elements.** For tiger beetles (specifically *Cicindela hybrida* (Linnaeus, 1767)) Evans (1965) referred to the large dorsal anterior occlusal teeth as incisors, and the posterior and more ventral complex of large teeth as molars. Kritsky and Simon (1995) used similar terms in their study of sexual dimorphism in mandibles of a wide selection of North American *Cicindela* (*sensu latissime*)species. The major elements of the occlusal surfaces of adult geadephagan mandibles were identified by Acorn and Ball (1991: 639-641, Figure 1) as terebra (with a distal incisor tooth, terebral ridge, and proximal terebral tooth) and retinaculum (with a distal anterior retinacular tooth, a double retinacular ridge, and proximal posterior retinacular tooth, or molar tooth. The retinaculum is posterior and ventral to the terebra. Here, based on similarity of position, we recognize the distal-most tooth as an incisor, the large dentiform projections along the occlusal margin as terebral teeth, and the posterior array of dentition as the retinaculum. See Table 2 for a complete list of the structural elements of mandibles and labrum-epipharynx. The abbreviations are used in the SEM figures to designate these structural elements.

**Table 2.** Terms and abbreviations for geadephagan mandibles (modified from Acorn and Ball 1991) and labrum-epipharynx (modified from Ball et al. 1995).

**Abbreviations**	**Terms**
**MANDIBLES**	
art	anterior retinacular tooth
B	base of mandible
bb	basal brush
bfb	basal face brush
irr	inferior retinacular ridge
it	incisor tooth
mss	multiple scrobal setae
mt	molar tooth
od	occlusal diastema
prt	posterior retinacular tooth
rc 1	retinacular tooth, cusp 1= art, in part
rc 2	retinacular tooth, cusp 2= art, in part
rc 3	retinacular tooth, cusp 3= prt, in part
rc 4	retinacular tooth, cusp 4= prt , in part
ret	retinaculum
rr	retinacular ridge
s	scrobe
srr	superior retinacular ridge
srt	supplementary retinacular tooth
ss	single scrobal seta
T	terebra
tr	terebral ridge
tt	terebral tooth
tt 1	terebral tooth 1
tt 1-1	terebral tooth 1, cusp 1
tt 1-2	terebral tooth 1, cusp 2
tt 1-3	terebral tooth 1, cusp 3
tt 2	terebral tooth 2
tt 3	terebral tooth 3
tt 4	terebral tooth 4
vg	ventral groove
vm	ventral microtrichia
**LABRUM-EPIPHARYNX**	
aps	anterior parapedial setae
epd	epipharynx, dorsal aspect
las	labral apical seta
ped	pedium
pp	parapedial projection
pps	posterior parapedial setae
pr	parapedial ridge
sc	sensillum coeloconicum

**Figure 1.**
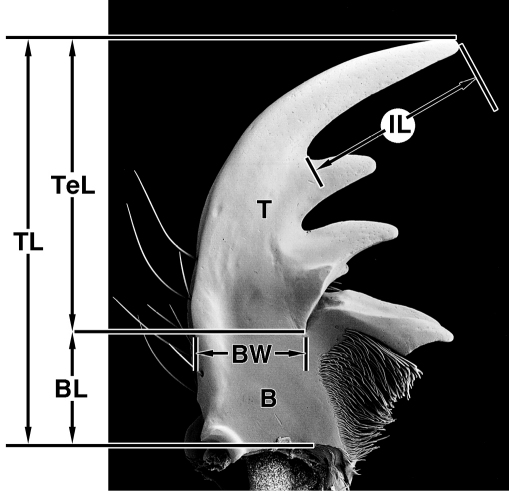
Measurements, plotted on photographic image of dorsal aspect of left mandible of *Amblycheila baroni* Rivers. Legend: **B** basal area; **BL** length of basal area; **BW** width of basal area; **IL** length of incisor tooth; **T** terebra; **TeL** length of terebra; **TL** total length.

**Measurements.** To assist in characterizing mandibles, four measurements (Figure 1) were taken and used to make ratios (Table 2). Being based on the figures of single specimens, differences in values of these ratios have no statistical significance. They are simply a means of standardizing descriptive statements.

**Descriptions.** Descriptions are brief, arranged in putative phylogenetic sequence, as reflected in the suprageneric taxa referred to in this paper.

## Descriptions And Comparisons

### Family Trachypachidae

**Classification.** Ranked as a family, this group of two genera and fewer than 10 species may be regarded as the adelphotaxon of the Carabidae (Kavanaugh 1998: 337; Maddison et al. 1999: 116, Figure 7; Dressler and Beutel 2010: Figure 22, p. 282 ), or as a group more closely related to the Hydradephaga (Acorn and Ball 1991: 645; Beutel 1998: 94, Figure 1, and p. 101).

**Exemplar taxon.** *Trachypachus gibbsii* LeConte, 1861.

Figures 2F, 5A–B

**Figure 2.**
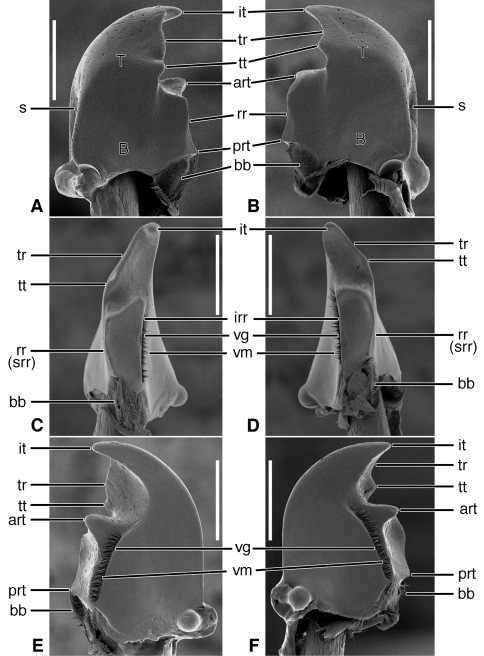
SEM photographs of mandibles of *Trachypachus gibbsi* LeConte. **A, C, E** left mandible, dorsal, occlusal, ventral aspects, respectively; **B, D, F** right mandible, dorsal, occlusal, ventral aspects, respectively. Legend: see Table 2. Scale bars = 0.2 mm.

**Structural features.** *Mandibles* (Figures 2A-F).Trigonal in dorso-ventral aspect, robust, basal width one half total length; curved downward slightly (Figures 2C-D). Lateral surface basally with scrobe broad, asetose, delimited by a dorsolateral and ventrolateral ridge. Terebra (**T**) short (terebral length one half total length), in occlusal aspect broad, with short incisor tooth (**it**) and with small terebral tooth (**tt**). Retinaculum slightly posteriad terebral tooth; short, broad, with two ridges (**rr** and **irr**), and two teeth (**art** and **prt**), anterior tooth near ventral margin (2C-D), posterior tooth dorsal. Basal face not evident. Basal brush (**bb**) small. Ventral surface smooth except for the ventral groove (Figures 2E-F, **vg**), extended anteriad anterior retinacular tooth, and with rather short and sparse microtrichia (**vm**).

*Labrum*(Figure 5A). Articulated with, not immovably attached to, clypeus, labral-clypeal suture evident. Distinctly transverse, sub-rectangular, row of 14 tactile setae (**las)** near anterior margin, on dorsal surface (Figure 5A). Form of anterior margin subtruncate, shallowly emarginate.

*Epipharynx* (Figure 5B). Pedium (**ped**) trianguloid, apex posterior, slightly arched, with a short parapedial projection (**pp**), margined laterally each side by a thin parapedial ridge (**pr**); parapedial ridge anteriorly each side curved to lateral margin of labrum as a short lateral arm. Anterior parapedial setae (**aps**) in a row anterior and parallel to lateral arms of parapedial ridge. Posterior parapedial setae (**pps**) in a row.

### Family Carabidae

Two major types of mandibles occur among the basal carabid lineages: the fluid-feeding *Ceroglossus* type, and the particulate-feeding *Pelophila* type, evidently depending upon manipulation of food (Evans and Forsythe 1985: 114). Associated with fluid-feeding, the labrum-epipharynx is immovably attached to the clypeus.

We identified two major types of epipharynx: the general one, shared with the Trachypachidae– pedium triangular in form, as described above; and the type confined to the tiger beetles– pedium broad, palatiform, markedly convex. For details, see below, under “Cicindelitae”.

### Supertribe Nebriitae, Tribe Pelophilini

**Classification.** This monogeneric group of two extant species is basal to the Supertribe Nebriitae, which in turn is a basal assemblage of the Carabidae (Kavanaugh 1998: 335, Figure 3, Maddison et al. 1999: 104, Figure 1).

**Exemplar taxon.** *Pelophila rudis* (LeConte, 1863).

Figures 3A-F, 5C-D

**Figure 3.**
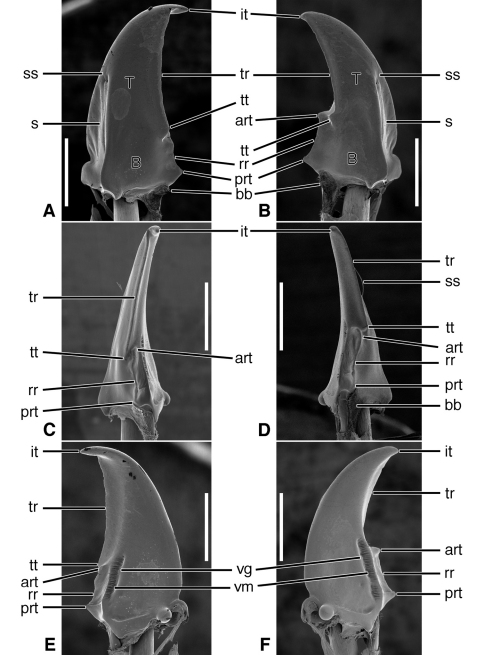
SEM photographs of mandibles of *Pelophila rudis* LeConte. **A, C, E** left mandible, dorsal, occlusal, ventral aspects, respectively; **B, D, F** right mandible, dorsal, occlusal, ventral aspects, respectively. Legend: see Table 2. Scale bars = 0.5 mm.

**Structural features.** *Mandibles* (Figures 3A-F).Trigonal in dorso-ventral aspect, robust, basal width one third total length; curved downward (Figures 3C-D). Lateral surface basally with scrobe broad, delimited by a dorsolateral and ventrolateral ridge; scrobe triangular, moderately deep, with single seta (**ss**). Terebra (**T**) long (terebral length two thirds total length), in occlusal aspect narrow, with short incisor tooth (**it**) and with small terebral tooth (**tt**). Retinaculum slightly posteriad terebral tooth, in dorso-ventral aspect (Figures 3A-B) narrow with single ridge (**rr**), and two teeth (**art** and **prt**), one at each end, in line with one another. Basal face not evident. Basal brush (**bb**) small. Ventral surface smooth except for the ventral groove (Figures 3E-F, **vg**), extended anteriad anterior retinacular tooth, and with rather short and dense microtrichia (**vm**).

*Labrum*(Figure 5C). Articulated with, but not immovably attached to clypeus, labral-clypeal suture evident. Distinctly transverse, sub-rectangular, row of five tactile setae (**las**) near anterior margin, on dorsal surface (Figure 5C). Form of anterior margin subtruncate.

*Epipharynx*(Figure 5D). Pedium (**ped**) broadly trianguloid, apex posterior, slightly arched, with a short parapedial projection (**pp**); margined laterally each side by a thin parapedial ridge (**pr**); parapedial ridge anteriorly each side curved to lateral margin of labrum as a short lateral arm. Anterior parapedial setae (**aps**) in a row anterior and parallel to lateral arms of parapedial ridge. Posterior parapedial setae (**pps**) few, in row along parapedial ridge.

**Comparisons.** The prominent retinaculum seems to be a feature of the Nebriitae (see Kavanaugh 1978: 856, Figures 54-58; and Acorn and Ball 1991: 647, 11A-D).

### Supertribe Carabitae, Tribe Ceroglossini

**Classification.** The Supertribe Carabitae is a basal lineage (Maddison et al 1999: 104, Figure 1; Kavanaugh 1998: 335, Figure 3). Erwin and Pearson (2008: 19) cite *Carabus* Linnaeus (meaning supertribe Carabitae, Erwin, personal communication 2011) as the adelphotaxon of the Cicindelitae; Liebherr and Will (1998: 142, Figure 57) include the Cicindelini as part of a quadritomy with three of the carabine tribes noted below.

The Carabitae includes 14genera, arranged in four tribes: Cychrini; Ceroglossini; Pamborini; and Carabini. The tribe Ceroglossini is monogeneric, including eightspecies.

**Exemplar taxon.** *Ceroglossus chilensis* (Eschscholtz, 1829).

**Figure 4.**
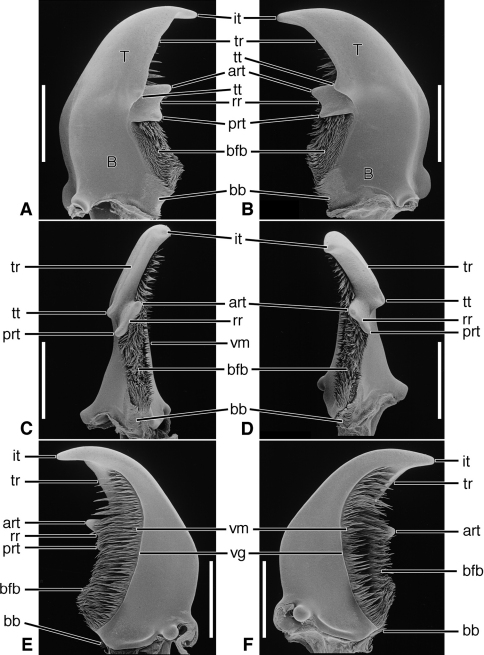
SEM photographs of mandibles of *Ceroglossus chilensis* Eschscholtz. **A, C, E** left mandible, dorsal, occlusal, ventral aspects, respectively; **B, D, F** right mandible, dorsal, occlusal, ventral aspects, respectively. Legend: see Table 2. Scale bars = 1.0 mm.

**Figure 5.**
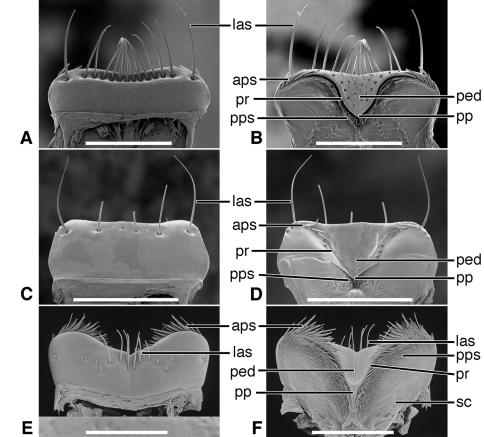
SEM photographsof labrum and epipharynx of: *Trachypachus gibbsi* LeConte (**A** labrum, dorsal aspect; **B** epipharynx, ventral aspect); *Pelophila rudis* LeConte (**C** labrum, dorsal aspect; **D** epipharynx, ventral aspect); *Ceroglossus chilensis* Eschscholtz (**E** labrum, dorsal aspect; **F** epipharynx, ventral aspect). Legend: see Table 2. Scale bars: **A–B** 0.2 mm; **C–D** 0.5 mm; **E–F** 1.0 mm).

**Plate 1.**
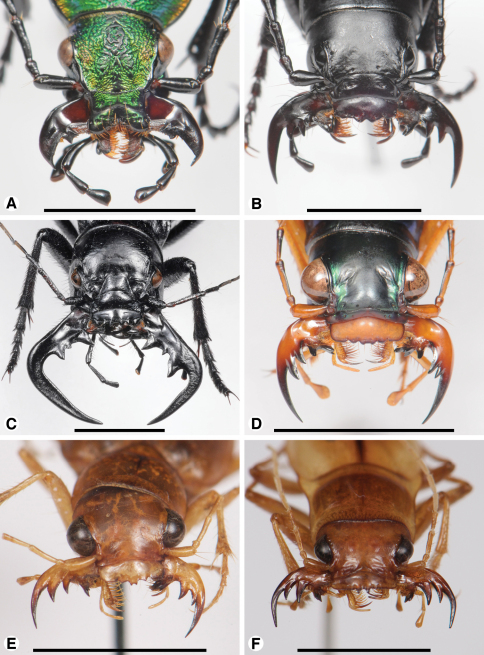
Digital images of head capsule, labrum, and mandibles dorso-frontal aspect, of: **A** *Ceroglossus chilensis* (Eschscholtz); **B** *Amblycheila baroni* (Rivers); **C** *Manticora mygaloides* Thomson; **D** *Megacephala regalis* Boheman; **E** *Phaeoxantha tremolerasi* (W. Horn); **F** *Platychile pallida* (Fabricius). Scale bars: **A, B, E, F** = 5 mm; **C, D** = 10 mm.

Figures 4A-F, 5E-F, Plate 1A

**Structural features.** *Mandibles*(Figures 4A-F, Plate 1A).Trigonal in dorso-ventral aspect (Figures 4A-B), robust, basal width one quarter total length; curved downward (Figures 4C-D). Lateral surface basally with scrobe broad, triangular, moderately deep, asetose, delimited by a dorsolateral and ventrolateral ridge. Terebra (**T**) long (terebral length only one half total length, but basal area exceptionally long), in occlusal aspect (Figures 4C-D) broad; with short incisor tooth (**it**) and with small terebral tooth (**tt**). Retinaculum slightly posteriad terebral tooth, short, oriented obliquely to long axis of mandible (Figures 4C-D), (broad single ridge (**rr**), and two teeth (**art** and **prt**), one at each end of terebral ridge, offset from one another. Basal face long, densely setose (**bfb**), setae seemingly continuous with microtrichia of ventral groove (Figures 4C-D). Basal brush (**bb**) small. Ventral surface smooth except for the ventral groove (Figures 4E-F, **vg**), extended anteriorly nearly to base of the incisor tooth, and with long and dense microtrichia (**vm**).

*Labrum*(Figure 5E, Plate 1A). Immovably attached to clypeus, labral-clypeal suture evident. Distinctly transverse, sub-rectangular, transverse cluster of numerous tactile setae (**las)** near anterior margin, on dorsal surface (Figure 5E). Form of anterior margin moderately deeply emarginate.

*Epipharynx*(Figure 5F, Plate 1A). Pedium (**ped**) rather narrowly trianguloid, apex posterior; slightly arched, with a long parapedial projection (**pp**); margined laterally each side by a thin parapedial ridge (**pr**); parapedial ridge anteriorly each side curved gradually to lateral margin of labrum. Anterior parapedial setae (**aps**) in a row anterior and parallel to lateral portion of parapedial ridge. Posterior parapedial setae (**pps**) rather numerous, in row along parapedial ridge.

**Comparisons.** The form of the mouthparts in *Ceroglossus* is in many ways reminiscent of that in cicindelites, and a more extensive survey of the Carabitae may well uncover additional shared features.

Another group of fluid feeders, the scaritines, was examined briefly, with inconclusive results. Some, (e.g. *Pasimachus* Bonelli, *Mouhotia* Laporte de Castelnau) appear not to possess a retinaculum, whereas in *Scarites* the retinaculum is either reduced and confluent with the base of the terebra, or the basal portion of the terebra is multidentate and vaguely reminiscent of what we interpret here as the retinaculum of cicindelites. Some evidence exists for a relationship between tiger beetles and scaritines (the “CPRS quartet” of Maddison et al. 1999, uniting tiger beetles, paussines, rhysodines, and scaritines); even these authors suggest that convergence seems a more likely explanation for this morphologically incongruous assemblage (but see Bell and Bell 1962 and Bell 1998 for evidence of a relationship between rhysodines and scaritines). Further study of this issue seems warranted (see, for example, Makarov 2008, who, based on morphological features, proposes to place the rhysodines and paussines in the suborder Archostemata).

### Supertribe Cicindelitae

**Classification.** Based principally on the phylogenetic conclusions of Pearson and Vogler (2001: 46, Figure 3.5) the tiger beetles are arranged here in five tribes: Amblycheilini; Manticorini; Megacephalini; Collyridini; and Cicindelini. Gálian et al. (2002: 1794, Figure 1) indicate the Megacephalini as polyphyletic, based on their study of multiple sex chromosomes in the cicindelites, the problem taxa being the oxycheiline genera *Oxycheila* and *Cheiloxya*. Based on their 18sRNA evidence and the DNA evidence of Vogler and Barraclough (1998: 255, Figure 1), these genera (and presumably *Pseudoxycheila*) belong in the tribe Cicindelini, and such a transfer obviates the taxonomic problem.

**Figure 6.**
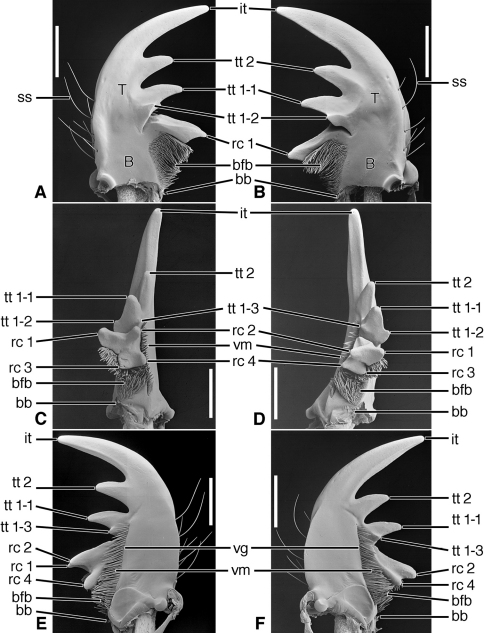
SEM photographs of mandibles of *Amblycheila baroni* Rivers. **A, C, E** left mandible, dorsal, occlusal, ventral aspects, respectively; **B, D, F** right mandible, dorsal, occlusal, ventral aspects, respectively. Legend: see Table 2. Scale bars 1.0 mm.

**Figure 7.**
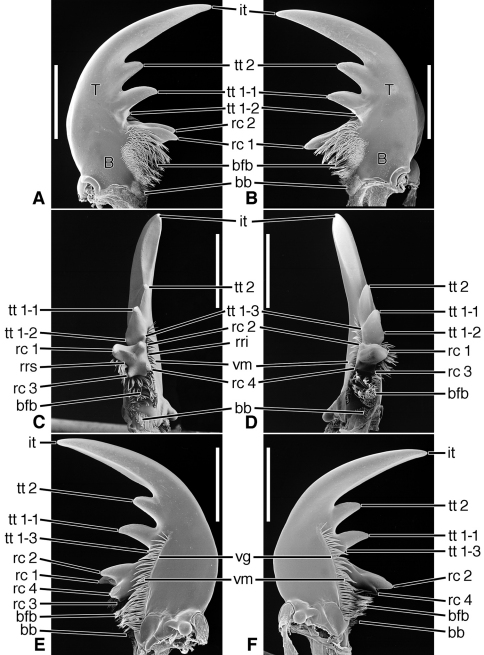
SEM photographs of mandibles of *Omus californicus* Eschscholtz. **A, C, E** left mandible, dorsal, occlusal, ventral aspects, respectively; **B, D, F** right mandible, dorsal, occlusal, ventral aspects, respectively. Legend: see Table 2. Scale bars = 1.0 mm.

**Figure 8.**
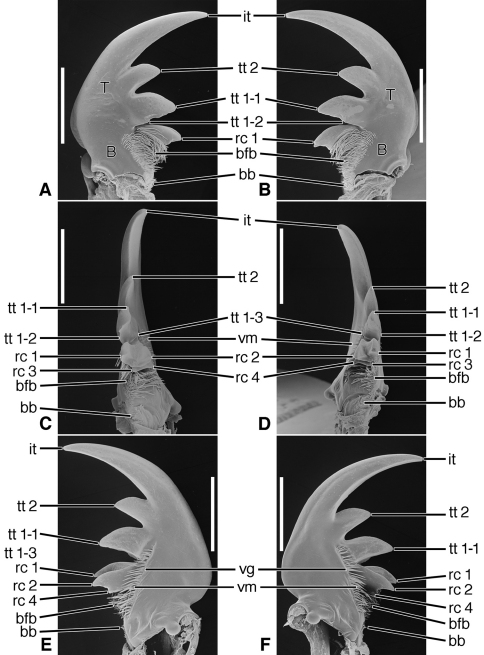
SEM photographs of mandibles of *Picnochile fallaciosa* Chevrolat. **A, C, E** left mandible, dorsal, occlusal, ventral aspects, respectively; **B, D, F** right mandible, dorsal, occlusal, ventral aspects, respectively. Legend: see Table 2. Scale bars = 1.0 mm.

**Structural Features.** *Mandibles* (Figures 6A-F–8A-F, 10A-F–12A-F, and 14A-F–16A-F).Trigonal in dorso-ventral aspect, robust to slender, basal width one fifth to one third total length; planar to curved downward, ventral curvature simple to complex. Lateral surface basally with scrobe broad, delimited by a dorsolateral and ventrolateral ridge, or with ventrolateral ridge only distinct, or lateral surface convex, without a scrobe; lateral surface asetose or multisetose (**ss**). Terebra (**T**) elongate (terebral length one half to three quarters total length), in occlusal aspect broad to slender, with long incisor tooth (**it**) (one third to one half total length), and with or without terebral teeth (**tt**) (in most taxa teeth two to three, variously prominent, unicuspidate or tricuspidate; in most taxa, sexually dimorphic). Retinaculum slightly posteriad basal terebral tooth or more widely separated by a distinct gap (the occlusal diastema, **od**; cf. Figures 12A, B, E, F). short, broad, with four or more cusps (**rc 1–4**); cusps of various sizes, in some taxa as large and prominent as terebral teeth. Basal face (**bf**) posteriad retinaculum, with long setae in form of a dense brush (**bfb**). Ventral surface smooth except for the ventral groove (**vg**) of various lengths, and with rather long and dense microtrichia (**vm**).

**Figure 9.**
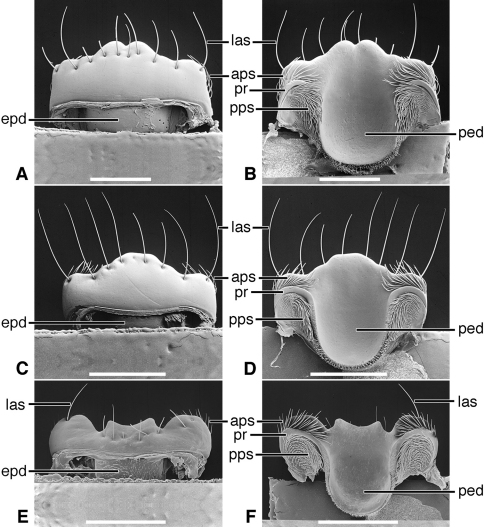
SEM photographsof labrum and epipharynx of: *Amblycheila baroni* Rivers (**A** labrum, dorsal aspect; **B** epipharynx, ventral aspect); *Omus californicus* Eschscholtz (**C** labrum, dorsal aspect; **D** epipharynx, ventral aspect); *Picnochile fallaciosa* Chevrolat (**E** labrum, dorsal aspect; **F** epipharynx, ventral aspect). Legend: see Table 2. Scale bars: 1.0 mm.

*Labrum* (Figures 9A, C, E; 13A, C, E; and 17A, C, E). Attached immovably to clypeus, although labral-clypeal suture evident. Of various proportions (Length/ Width 0.20-1.02), i.e., distinctly transverse, sub-rectangular, to slightly longer than wide and trapezoid or trianguloid; row of tactile setae either near anterior margin, on dorsal surface (Figure 9A), or on apical margin (Figure 13E). Form of anterior margin various, from subtruncate (Figure 13C) to simply projected medially (Figure 9C), to markedly projected (Figure 17C); projection one or several denticles, or broadly rectangular (Figure 9C). See also Cazier (1954: 306-307, Figures 124-169).

*Epipharynx* (Figures 9B, D, F; 13B, D, F; and 17B, D, F).Pedium (**ped**) palatiform, markedly arched, ventral surface concave; posteriorly broadly rounded, without a parapedial projection, margined laterally each side by a thin parapedial ridge (**pr**); parapedial ridge anteriorly each side curved to lateral margin of labrum as a short lateral arm. Parapedial setae in a row or cluster anterior (**aps**) to or posterior (**pps**) to lateral arms of parapedial ridge.

**Comments.** Cicindelite mandibles are readily distinguishable from those of other geadephagans by a combination of complex and distinctive retinacular structure (essential to the “pre-oral mill” of Evans 1965: 64) and multi-toothed terebra. Among the tiger beetle tribes, the more plesiotypic manticorines and amblycheilines exhibit greater mandibular robustness compared to the more apotypic megacephalines, collyridines and cicindelines, and most taxa with either two or three terebral teeth, this number reduced to one in many collyridines (especially on the left mandible) and in some cicindelines (e.g. *Therates*), but increased to as many as four in other collyridine lineages and to five in some cicindeline lineages. The labrum-epipharynx is generally short in the amblycheilines, manticorines, and megacephalines, and sub rectangular or elongate and dorsally convex (elongation presumably evolving several times) among the collyridines and cicindelines.

### Supertribe Cicindelitae: Tribe Amblycheilini

**Synonymic note.** Commonly known as the Omini W. Horn (1907: 466) (for example, Ball and Bousquet 2001: 71; Pearson and Vogler: 2001: 48), in fact the correct name is Amblycheilini Csiki (1903: 124), based on the principle of priority. For details, see Madge (1989: 460 and 466).

**Classification.** This tribe includes three Western Hemisphere genera: the western Nearctic *Amblycheila* Say and *Omus* Dejean; and the southern Neotropical *Picnochile* Motschulsky (female). If the southern Afrotropical monobasic genus *Platychile* Macleay is placed in this group (e.g. Pearson and Vogler 2001: 48), the tribal name becomes Platychilini W. Horn (1893: 325) (Madge 1989: 460 and 466), but see below for our reasons for not choosing this arrangement.

**Exemplar taxa.** *Amblycheila baroni* (Rivers, 1890) (female); *Omus californicus* (Eschscholtz, 1829) (male); *Picnochile fallaciosa* (Chevrolat, 1854) (female).

Also examined but not treated in detail were two males of *Platychile pallida*(Fabricius, 1801).

**Figure 10.**
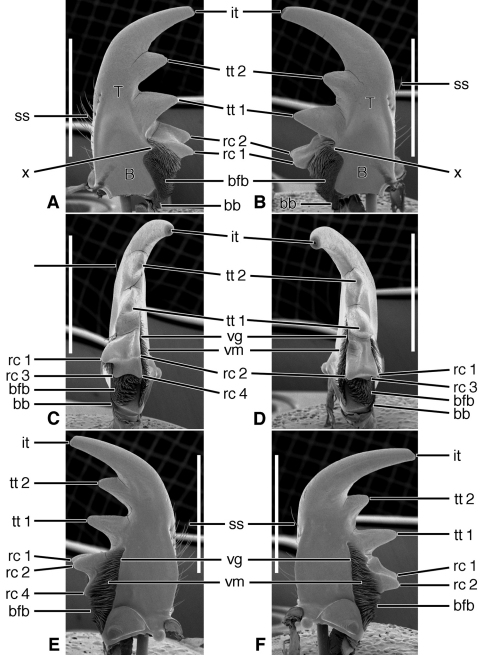
SEM photographs of mandibles of *Manticora latipennis* Waterhouse. **A, C, E** left mandible, dorsal, occlusal, ventral aspects, respectively; **B, D, F** right mandible, dorsal, occlusal, ventral aspects, respectively. Legend: see Table 2. Scale bars = 5.0 mm.

**Structural features.** *Mandibles*(Figures 6A-F – 8A-F; Plate 1B).— With mandibular features of Cicindelitae, restricted as follows. Values for ratios BW/TL. TeL/TL, and IT/TL as in Table 3; planar (Figures 6C-D) to moderately curved ventrad (Figures 7C-D). Lateral surface basally with scrobe broad, delimited by a dorsolateral and ventrolateral ridge;scrobe multisetose (Figures 6A-F, **ss**) or glabrous. Diastema absent. Terebral teeth two, terebral tooth 1 tricuspidate (Figures 6A-F, **tt 1-1**, **tt 1-2**, **tt 1-3**). Retinacular cusps (Figures 6C-D) **rc 1** and **rc 2** directly opposite one another, also **rc 3** and **rc 4** directly opposite one another.Ventral groove (Figures 6E-F and 10E-F, **vg**)moderately long, extended about to middle of terebral tooth 1.

**Table 3.** Ratios for features of left mandible of exemplar specimens of *Trachypachus*, *Pelophila*, *Ceroglossus*, and nine genera of Cicindelitae, representing Tribes Amblycheilini, Manticorini, Megacephalini, Collyridini, and Cicindelini.

**Taxon**	**BW^2^/TL^1^**	**TeL^3^/TL**	**IL^4^/TL**
*Trachypachus gibbsii* LeConte	0.47	0.53	0.12
*Pelophila rudis* LeConte	0.32	0.76	0.10
*Ceroglossus chilensis* Eschscholtz	0.24	0.54	0.12
*Amblycheila baroni* (Rivers)	0.27	0.77	0.46
*Omus californicus*	0.27	0.59	0.57
*Picnochile fallaciosa* (Chevrolat)	0.32	0.71	0.43
*Manticora tuberculata* (DeGeer)	0.27	0.68	0.33
*Tetracha carolina* (Linnaeus)	0.30	0.70	0.34
*Pogonostoma chalybeum* (Klug)	0.20	0.69	0.42
*Therates basalis* Dejean	0.19	0.66	0.37
*Oxycheila* species	0.24	0.70	0.39
*Cicindela longilabris* Say	0.17	0.68	0.45

^1^ Total Length; ^2^ Basal width; ^3^ Terebral Length; ^4^ Incisor Length

*Labrum*(Figures 9A, C, E; Plate 1B). Transverse (L/W 0.20-0.36), in form rectanguloid. Anterior margin distinctly projected medially, otherwise various: projection truncate (Figure 9C) or narrowly notched (Figure 9A); or anterior margin with two paramedial notches, anterior margin of medial projection broadly emarginate (Figure 9E). Single preapical row of 8-10 setae (**las**).

*Epipharynx* (Figures 9B, D, F). Row of anterior parapedial setae (**aps**) extended laterally, parallel to lateral extensions of parapedial ridge (**pr**).

**Comments.** Gissler (1879: 234) postulated that the genus *Amblycheila* represented the “lowest.....and certainly the oldest line of descent...that probably diverged in the Mesozoic age”- a remarkably prescient line of thought, considering that it was developed more than a century ago.

The genus *Platychile* deserves special comment here. We examined two males of *Platychile pallida* (Fabricius) using light microscopy. A number of mandibular features (planar, markedly curved; terebra bidentate; retinaculum quadridentate) place the genus among the more basal lineages. On the other hand, the mandibles are so flat as to virtually eliminate the scrobe (and are thus reminiscent of the more derived tiger beetles), and they possess a single cusp terebral tooth 1 instead of the three cusps characteristic of the Western Hemisphere genera Plate 1F). The rectangular labrum (Plate 1F) has an anterior margin with two paramedial dentiform projections, flanked each side by two more short and blunt projections (six, in all) and six setae on the anterior (not apical) surface. The labrum, in fact, in form and setation, is strongly reminiscent of that of the tribe Megacephalini. Further, the body size and form is not unlike that of what could be expected in the megacephaline genus *Phaeoxantha*. The color pattern is also megacephaline-like, and a careful reading of Pearson and Vogler (2001: 53-57) indicates that such a feature may have importance in tiger beetle evolution. The unusual habitus of *Platychile* may also derive in part from convergence or mimicry, since these nocturnal beetles show a consistent ecological association with the diurnal *Eurymorpha cyanipes* (Hope, 1838) (Werner 2000), with which they share an oval dorsoventrally flattened appearance, without pronounced elytral humeri—a resemblance that seems unlikely to stem from mere coincidence. *Platychile* may be the adelphotaxon of the Western Hemisphere genera (Galián, et al. 2002: 1794, Figure 1), or it could be treated as a monobasic group of uncertain affinity, our preferred arrangement here.

### Supertribe Cicindelitae: Tribe Manticorini

**Classification.** This tribe includes the southern Afrotropical genera *Manticora* Fabricius, 1792 and *Mantica* Kolbe, 1896.

**Exemplar taxon.** *Manticora tuberculata* (DeGeer, 1778) (female).

Other specimens examined: see Appendix.

**Structural features.** *Mandibles*(Figures 10A-F). With mandibular features of Cicindelitae, restricted as follows. Values for ratios BW/TL. TeL/TL, and IT/TL as in Table 3. Robust; markedly curved ventrad (Figures 10C-D). Lateral surface basally with scrobe broad, delimited by a dorsolateral and ventrolateral ridge;scrobe multisetose (Figures 10A-F, **ss**). Diastema absent. Terebral teeth two (some female *Manticora*) or three (male *Manticora*, somefemale *Manticora*, andboth sexes of *Mantica*), terebral tooth 1 monocuspidate (Figures 10A-F, **tt 1**); or bicuspidate, **tt 1-2 (**appearing separate from **tt 1-1** in genus*Mantica*. Retinacular cusps (Figures 10C-D) **rc 1** and **rc 2** directly opposite one another, also **rc 3** and **rc 4** directly opposite one another.Ventral groove (Figures 10E-F, **vg**)moderately long, extended about to middle of terebral tooth 1.

The mandibles of *Manticora* are pronouncedly sexually dimorphic, larger in males and asymmetric in form, the right mandible typically exhibiting greater elongation of the incisor region than the left. In *Mantica*, sexual dimorphism is slight, and some but not all males show larger left than right mandibles (Franzen and Heinz 2005: 299).

*Labrum* (Figure 13A). Transverse (L/W 0.20-0.36), in form rectanguloid. Anterior margin distinctly crenate, *Manticora* with six teeth, *Mantica* with four teeth (Franzen and Heinz 2005: 300), median projection short, broad, emarginate anteriorly. Single preapical row of 10 setae (**las**).

*Epipharynx*(Figure 13B).Row of anterior parapedial setae (**aps**) extended laterally, parallel to lateral extensions of parapedial ridge (**pr**).

**Comments.** See also Plate 1C. In structure of mandibles and labrum-epipharynx, the Manticorini seems most similar to the Amblycheilini. However, the marked ventrad curvature of the mandibles is suggestive of the more derived megacephalines, cicindelines and collyrines. With the genera *Amblycheila* and *Pogonostoma*, members of *Manticora* share multisetose scrobes.

For details about way of life, classification and relationships of *Manticora*, see Oberprieler and Arndt (2000). Franzen and Heinz (2005) provide a valuable review, including illustrations of mandibles, of the monobasic genus *Mantica* (type species, *Mantica horni* Kolbe, 1896).

### Supertribe Cicindelitae: Tribe Megacephalini

**Classification.** This tribe includes more than 100 species, arrayed in eight genera (Zerm et al. 2007; and Huber 1994). Galián et. al. (2002: 1794) indicate that, based on 18sRNA analysis the Megacephalini is polyphyletic, with the genera *Cheiloxya* and *Oxycheila* sharing a closer relationship with the cicindelines than with the megacephalines. Vogler and Barraclough (1998: 256, Figure 2) had indicated that DNA evidence showed the same thing for *Oxycheila*. No doubt, *Oxycheila*, *Cheiloxya* and putative close relative *Pseudoxycheila* belong in the tribe Cicindelini.

**Exemplar Taxon.** *Tetracha carolina* (Linnaeus, 1767) (male).

Other specimens examined: see Appendix.

**Figure 11.**
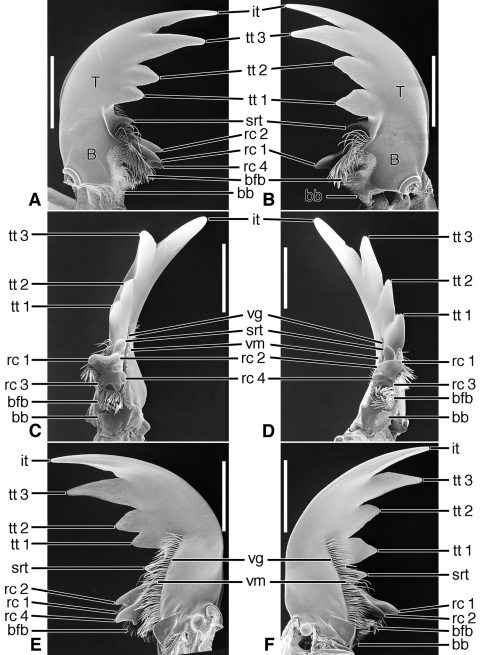
SEM photographs of mandibles of *Tetracha carolina* Linnaeus. **A, C, E** left mandible, dorsal, occlusal, ventral aspects, respectively; **B, D, F** right mandible, dorsal, occlusal, ventral aspects, respectively. Legend: see Table 2. Scale bars = 1.0 mm.

**Structural Features.** *Mandibles* (Figures 11A-F).With mandibular features of Cicindelitae, restricted as follows. Values for ratios BW/TL. TeL/TL, and IT/TL as in Table 3. Robust; markedly curved ventrad (Figures 11C-D). Lateral surface basally with scrobe broad, delimited by a dorsolateral and ventrolateral ridge;scrobe glabrous (Figures 11A-F). Diastema absent. Terebral teeth three, terebral tooth 1 monocuspidate (Figures 11A-F, **tt 1**). Retinaculum with supplementary tooth anteriorly (**srt**) in *Tetracha* and *Megacephala*, very small in *Phaeoxantha*. Retinacular cusps (Figures 11C-D) **rc 1** and **rc 2** directly opposite one another, also **rc 3** and **rc 4** directly opposite one another.Ventral groove (Figures 11E-F, **vg**)moderately long, extended about to base of terebral tooth 2.

**Figure 12.**
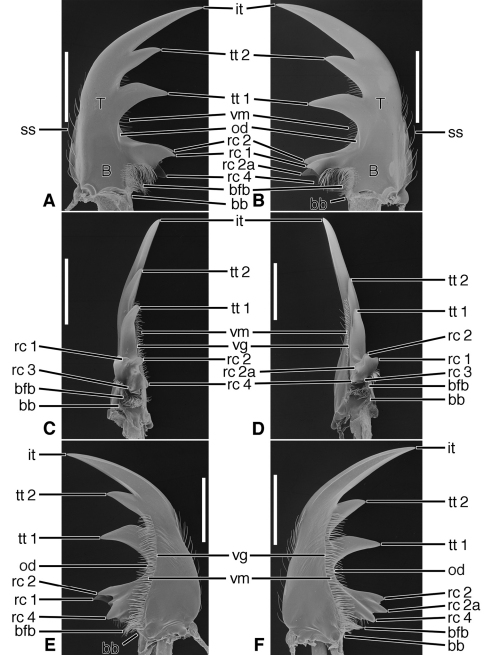
SEM photographs of mandibles of *Pogonostoma chalybeum* Klug. **A, C, E** left mandible, dorsal, occlusal, ventral aspects, respectively; **B, D, F** right mandible, dorsal, occlusal, ventral aspects, respectively. Legend: see Table 2. Scale bars = 1.0 mm.

**Figure 13.**
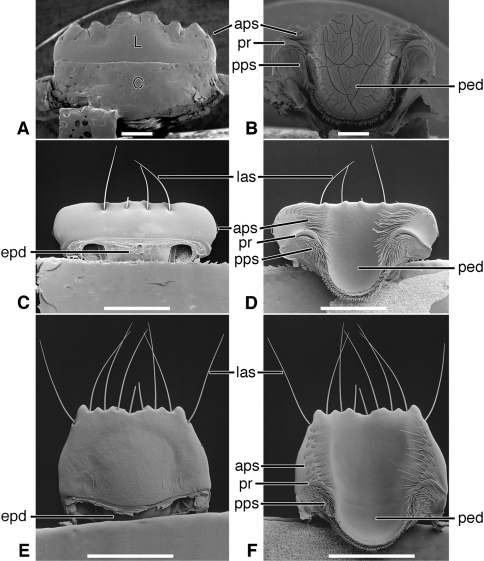
SEM photographsof labrum and epipharynx of: *Manticora latipennis* Waterhouse (**A** labrum, dorsal aspect; **B** epipharynx, ventral aspect); *Tetracha carolina* Linnaeus (**C** labrum, dorsal aspect; **D** epipharynx, ventral aspect); *Pogonostoma chalybeum* Klug (**E** labrum, dorsal aspect; **F** epipharynx, ventral aspect). Legend: see Table 2. Scale bars: 1.0 mm.

*Labrum* (Figure 13C). Transverse (L/W 0.28), in form rectanguloid. Anterior margin shallowly crenate, medially, median projection short, broad, emarginate anteriorly). Single preapical row of four setae (**las**).

*Epipharynx* (Figure 13D). Row of anterior parapedial setae (**aps**) extended anteriorly, at about right angle to lateral extensions of parapedial ridge (**pr**).

**Comments.** See also Plate 1D, E. The long terebral tooth 3 illustrated for the specimen of *Tetracha carolina* seems to be characteristic of males of that genus, contrasting markedly with the shorter **t3** of the corresponding females. Number of labral setae in Megacephalini ranges from four to seven.

### Supertribe Cicindelitae: Tribe Collyridini

**Classification.** This group is the equivalent of and co-extensive with Walther Horn’s (1908) “Phylum” Alocosternalia. This tribe includes seven genera arranged in two subtribes: Ctenostomatina, and Collyridina (Lorenz 2005: 22).

**Exemplar taxon.** *Pogonostoma chalybeum* (Klug, 1835) (male).

Other collyridines examined include representatives of subtribes Ctenostomatina and Collyridina. See appendix.

**Structural features.** *Mandibles* (Figures 12A-F). With mandibular features of Cicindelitae, restricted as follows. Values for ratios BW/TL, TeL/TL, and IT/TL as in Table 3. Slender; markedly curved ventrad (Figures 12C-D). Lateral surface basally with scrobe broad, delimited by a dorsolateral and ventrolateral ridge;scrobe multisetose (Figures 12A-F, **ss**). Diastema (**od**) present. Terebral teeth various in size and number (see “Variation” below, for details). Retinaculum without or with (*Collyris* only) supplementary tooth anteriorly (**srt**). Retinacular complex large, cusps (Figures 12C-D) diagonally arranged, number of cusps various (see below for details)Ventral groove (Figures 12E-F, **vg**)moderately long, extended about to base of terebral tooth 2.

*Labrum* (Figure 13E). Elongate (L/W 0.63), in form trapezoidal. Anterior margin shallowly crenate. Single apical row of nine setae (**las**), each seta inserted in base of crenulation. For details, See “Variation”, below.

*Epipharynx* (Figure 13F). Pedium (**ped**) markedly concave. Row of anterior parapedial setae (**aps**) extended anteriorly, at about right angle to lateral extensions of parapedial ridge (**pr**). For details, See “Variation”, below.

**Variation.** Terebral teeth two (each mandible), **tt 1** monocuspidate (Figures 12A-F) (*Pogonostoma*), or only one tooth on each mandible (*Collyris*); or terebral teeth asymmetric, with two on right mandible and one on left mandible (*Ctenostoma*), or one on left mandible, two on right mandible (*Tricondyla*). Retinacular cusps five each mandible (*Pogonostoma*), or seven on left mandible, five on right mandible (*Ctenostoma*), or five on left mandible, four on right mandible (*Tricondyla* and *Collyris*).

Although the labrum-epipharynx is consistently elongate and dorsally convex,the number of anterior marginal teeth varies from five to eight, some taxa with medial crenulation and an odd number of teeth; some with median notch and an even number of teeth; lateral pair of teeth generally acute, median teeth in form of rounded crenulations; shallow grooves present, in some taxa on labrum and/ or epipharynx, extended posteriad notch separating lateral and medial teeth. Number of labral setae various, from six to 14. Anterior parapedial setae extended almost to anterior margin in *Pogonostoma*, but less so in other collyridine taxa. It is not clear how medial teeth, notches, and/or setae have evolved from their paired bilateral homologues.

**Plate 2.**
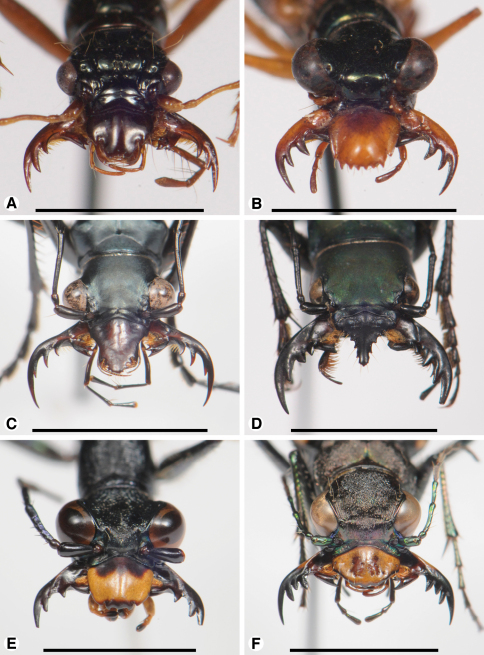
Digital images of head capsule, labrum, and mandibles dorso-frontal aspect, of: **A** *Ctenostoma ichneumoneum*Dejean; **B** *Therates erinnys*Bates; **C** *Cheiloxya binotata* Laporte de Castelnau; **D** *Pseudoxycheila* species?; **E** *Dromica junodi* Péringuey; **F** *Cicindela longilabris* Say. Scale bars: **A** = 3 mm; **B, E** =4 mm; **C, D, F** = 5 mm.

**Comments.** See Plate 2A for illustrations of the mandibles and labrum of *Ctenostoma ichneumoneum* Dejean, 1833. Clearly, although the collyridines present a diversity of mouthpart configurations, there is no obvious reason to doubt the use of *Pogonostoma* as an exemplar for the group, likely to exhibit a more or less plesiotypic structural condition. The ant-like body form of most collyridines may have constrained the head shape and therefore mouthpart structure of these beetles to some extent, but this is merely conjecture on our part.

### Supertribe Cicindelitae: Tribe Cicindelini

**Classification.** This tribe includes more than 1500 species, arranged in five subtribes: Theratina; Oxycheilina; Iresina; Prothymina; and Cicindelina (Vogler and Barraclough, 1998).

**Exemplar taxa.** *Therates basalis* Dejean (Theratina); *Oxycheila* species (Oxycheilina); and *Cicindela longilabris* Say (Cicindelina).

Other cicindelines examined: see Appendix for names;plus numerous species of Nearctic *Cicindela*, principallyfor form, and for number of labral setae.

**Figure 14.**
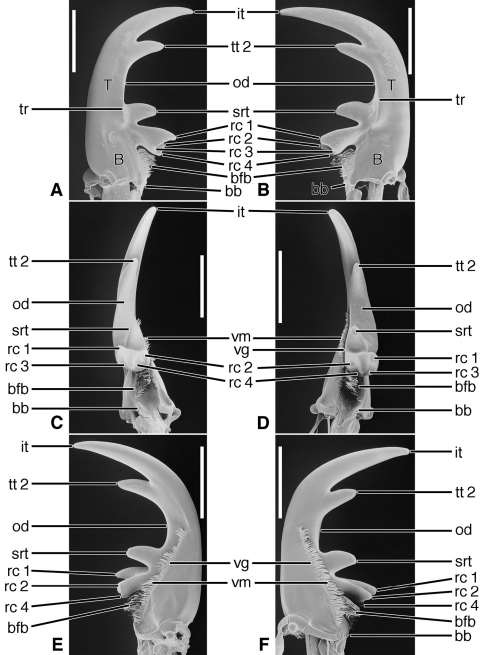
SEM photographs of mandibles of *Therates basalis* Dejean. **A, C, E** left mandible, dorsal, occlusal, ventral aspects, respectively; **B, D, F** right mandible, dorsal, occlusal, ventral aspects, respectively. Legend: see Table 2. Scale bars = 1.0 mm.

**Figure 15.**
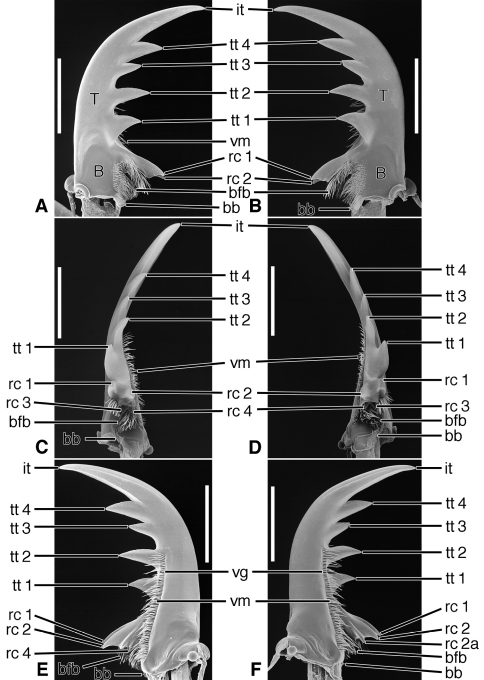
SEM photographs of mandibles of *Oxycheila* species.Eschscholtz. **A, C, E** left mandible, dorsal, occlusal, ventral aspects, respectively; **B, D, F** right mandible, dorsal, occlusal, ventral aspects, respectively. Legend: see Table 2. Scale bars = 1.0 mm.

**Figure 16.**
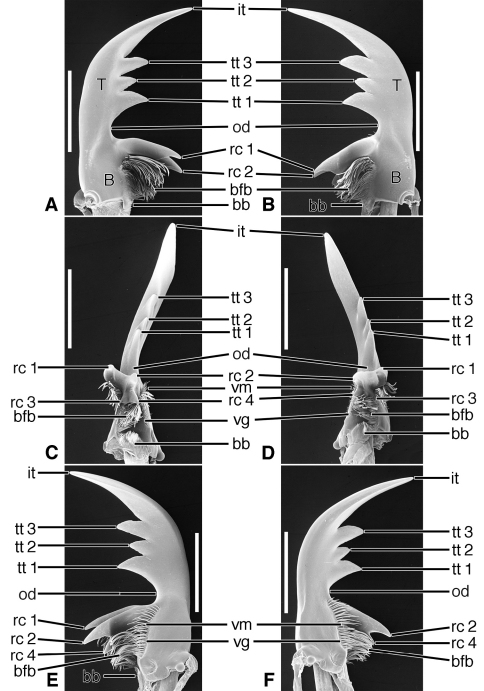
SEM photographs of mandibles of *Cicindela longilabris* Say. **A, C, E** left mandible, dorsal, occlusal, ventral aspects, respectively; **B, D, F** right mandible, dorsal, occlusal, ventral aspects, respectively. Legend: see Table 2. Scale bars = 1.0 mm.

**Structural features.** *Mandibles* (Figures 14A-F- 16A-F; Plate 2F). With mandibular features of Cicindelitae, restricted as follows. Values for ratios BW/TL, TeL/TL, and IT/TL as in Table 3. Slender; markedly curved ventrad (Figures 14C-D). Lateral surface basally without scrobe, or scrobe very narrow, confined to lateroventral surface (*Dromica*), and without setae. Diastema present (Figures 14A-F and 16A-F, **od**) or absent (Figures 15A-F). Terebral teeth various in size and number: one, (Figures 14A-F, **tt 2**) to four (Figures 15A-F, **tt 1 – tt 4**),with a maximum of five, observed in *Oxygonia gloriola* Bates, 1872. Retinaculum without or with (*Therates*, Figures 14A-F) supplementary tooth anteriorly (**srt**). Retinacular complex large, cusps diagonally arranged, number of cusps four on each mandible (**rc 1- rc 4**).Ventral groove (Figures 15E-F, **vg**)moderately long, extended about to base of terebral tooth 2, or shorter, extended only to base of retinacular complex (Figures 16E-F).

**Figure 17.**
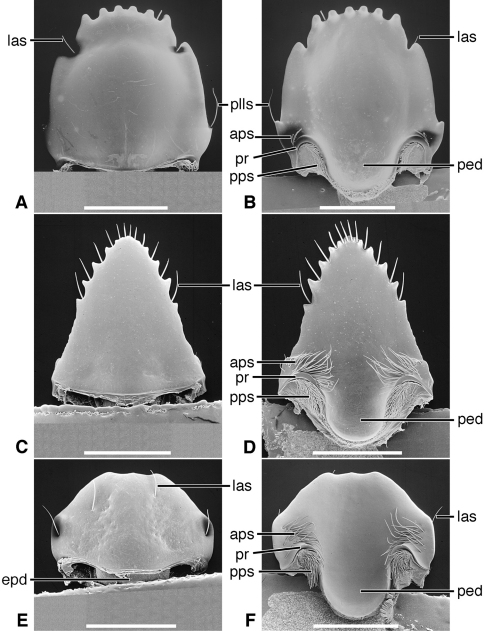
SEM photographsof labrum and epipharynx of: *Therates basalis* Dejean (**A** labrum, dorsal aspect; **B** epipharynx, ventral aspect); *Oxycheila* species (**C** labrum, dorsal aspect; **D** epipharynx, ventral aspect); *Cicindela longilabris* Say (**E** labrum, dorsal aspect; **F** epipharynx, ventral aspect). Legend: see Table 2. Scale bars: 1.0 mm.

*Labrum* (Figures 17A, C, E). Markedly varied. Illustrations as follows. Form transverse (L/W 0.41, Figure 17E), as long as wide (L/W 1.00, Figure 17A), or slightly longer than wide (L/W 1.02, Figure 17C). Trapezoidal to trianguloid. Anterior margin nearly smooth or distinctly crenate. Labral setae (**las, plls**) four to 12, on dorsal or apical surface, inserted at base of crenations.

*Epipharynx*(Figures 17B, D, F). Anterior parapedial setae (**aps**) in row parallel to lateral arm of parapedial ridge (Figure 17D), extended in short row anterior and obliquely to lateral arm of parapedial ridge (Figure 17E) or only very few setae laterally (Figure 17B).

**Variation.** For illustrations of mandibles and labra of additional cicindeline taxa, see Plate 2C-E. The mandibles of three exemplar taxa described above, each representing a different cicindeline subtribe, differ strikingly from one another, seeming to indicate an appreciable level of divergence in this tribe. Within the subtribe Cicindelina, Kritsky and Simon (1995) showed that the mandibles of various Nearctic species of *Cicindela* exhibit more or less striking sexual dimorphism, the number of terebral teeth being constantly three, but differing in relative size. Similarly, Satoh and Hori (2004: 211) showed sexual dimorphism in the Palaearctic species, *Lophyridia angulata* (Fabricius, 1781), as did Oberprieler and Arndt (2000: 86) for *Manticora* adults, and Franzen and Heinz (2005: 299) for *Mantica* adults. Although not well studied, it appears that in all but a few aberrant individuals, the left mandible adducts above the right (“left-superior chirality”; Richardson 2010).

In their remarkable study of geographical variation in *Cicindela dorsalis* Say, 1817, Boyd and Rust (1982: 225, 229) described a dentiform projection (the “submandibular tooth”) on the ventral terebral surface of the right mandible of males, only. This projection, of unknown function, was shown to vary in size (their paper, p. 228, Figure 6) depending upon subspecies which, in turn, was correlated with overall body size.

Intensive investigation of mandibular length (Ganeshaiah and Belavadi 1986; Mury Meyer 1987; Niemelä and Ranta 1993; Pearson 1980; Pearson and Juliano 1991; Pearson and Mury 1979; Satoh et al. 2003; and Satoh and Hori 2004) has shown that this factor is important in the structuring of tiger beetle communities, principally through resource partitioning (Pearson and Vogler 2001: 198-203).

Our three exemplar taxa differ markedly from one another in form and setation of the labrum. We note that the insertion of the labral setae on the dorsal surface of the labrum in *Cicindela longilabris* Say, 1824 (Figure 17E) is a relatively basal condition. Cazier (1954: 2306-309, Figures 129-223), in his treatment of the Mexican species of *Cicindela*, illustrated striking differences in labra, particularly in form of the anterior margin of the labrum, proportions, and number of dorsal setae (from four to more than 30).

The epipharynges of the three exemplar taxa are basically similar to one another, but differ in the anterior parapedial setation. The small number of such setae exhibited by *Therates basalis* Dejean, 1826 (Figure 17B) is the most derived, and is similar to that of the collyridine, *Ctenostoma metallicum* Laporte de Castelnau, 1834 (not illustrated).

## Evolution

**Figure 18.**
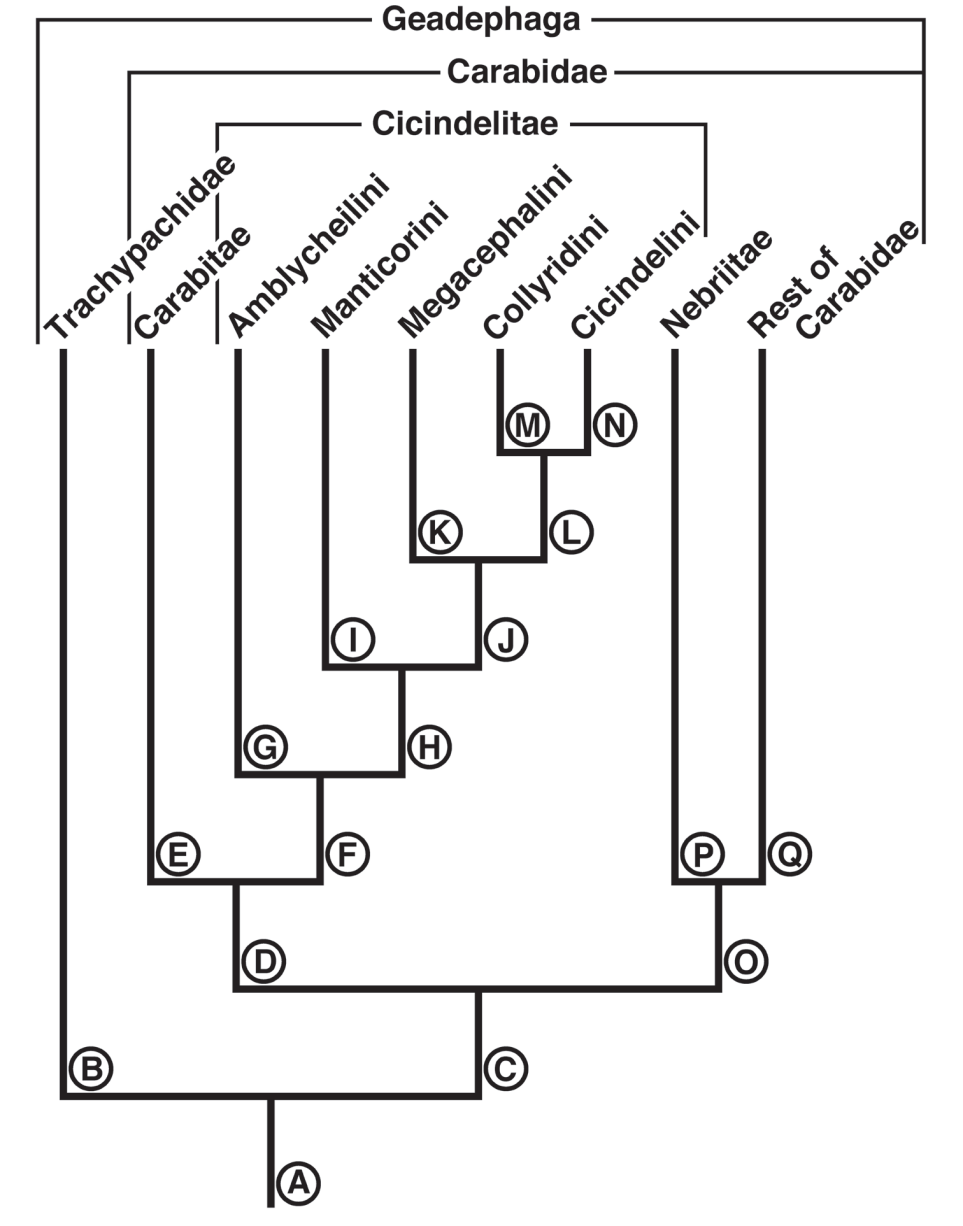
Diagram of a reconstructed phylogeny of the cicindelite tribes, related higher carabid taxa, and Trachypachidae. Reference points on this diagram are the lineages indicated by the capital letters **A** to **Q**.

The following hypothesis of mandibular and labral-epipharyngeal evolution is illustrated in the reconstructed phylogeny (Figure 18) based on Vogler and Barraclough (1998) and largely corroborated by other studies (Liebherr and Will 1998; Gálian et al. 2002), although the latter study places the Manticorini as the adelphotaxon of the Amblycheilini. Reference points on this diagram are the lineages indicated by the capital letters **A** to **P**.

Lineage **A** represents the common ancestor of the Geadephaga, in which we assume the mandibles possessed a distinct terebra and retinaculum, of unknown form, but probably *Pelophila*-like. The labrum-epipharynx was likely movably articulated with the head capsule.

Lineage **B** represents the evolution of the Trachypachidae, with mandible (Figures 2A-F) and labral-epipharynx (Figures 5A-B) features as follows: mandibles, short, broad, slightly curved ventrally, terebra relatively short, with short incisor and short terebral teeth, a very short basal face, and the basal brush (**bb**) serving as the basal face setae. The retinaculum includes two long retinacular ridges (**rr**, **srr**), and is bicuspidate, teeth (**art**, **prt**) short.

The labrum-epipharynx is movably articulated with the head capsule: labrum transverse, rectanguloid, anterior margin subtruncate, dorsal surface with a row of numerous setae (14, more or less); epipharynx with short, trianguloid pedium, with few anterior and posterior parapedial setae, and a small parapedial projection. Diet yet to be determined: probably partially fluid and partially particulate matter.

Lineage **C** The stem of the Carabidae. Evolution of the mandibles includes only simplification of the retinaculum to a single broad ridge and possibly lengthening of the terebra. The diet was probably generalized, including both solid and fluid food (the “mixed feeders” of Forsythe 1983: 371).

Lineage **D** Evolution of carabite-cicindelite mandibles as the diet became fluid, only, the food principally soft-bodied invertebrates. Includes development of a somewhat enlarged and densely setose basal face for retention of the fluid component of prey tissues for extra-oral digestion, and the labrum-epipharynx becoming immovably attached to the head capsule.

Lineage **O** Evolution of mandibles (Figures 3A-F) of the nebriite- “rest of Carabidae” Lineage includes development of a scrobal seta (**ss**), and narrowing of the single ridged retinaculum (**rr**). (Not followed further here, but see Acorn and Ball 1991).

Lineage **E** Evolution of mandibles of Carabitae (Figures 4A-F) includes marked lengthening of the basal face (**bf**) shortening of the retinaculum and its diagonal orientation (**rr**). The ventral groove (Figures 4E-F, **vg**) became markedly lengthened to nearly the base of the incisor tooth, and the ventral microtrichia (**vm**) became markedly lengthened. The anterior margin of the labrum (Figure 5E) became markedly emarginate.

Lineage **F** Evolution of mandibles of Cicindelitae includes hypertrophy of the incisor tooth, development of a second terebral tooth, hypertrophy of the terebral teeth, and the scrobes becoming multisetose. But the most striking mandibular development is seen in the retinaculum, which becomes markedly enlarged and quadricuspidate – an important element of the preoral mill.

The labrum becomes lengthened, its anterior margin sinuously arched and sparsely setose, the number of anterior setae reduced to 8-10. The epipharynx is extensively modified, becoming palatiform, dorsally arched, and widened posteriorly, with loss of the parapedial projection. Evans and Forsythe (1985: 116) describe the mode of feeding, unique among fluid-feeding carabids, in which prey is held, punctured and sheared by the incisor and terebral teeth, then passed posteriorly by the maxillary lacinia to the crushing teeth of the retinaculum. Within the preoral mill (bordered dorsally by the epipharynx, ventrally by the setose labium and laterally by the setose retinacular region of the mandibles), a food bolus is rotated posterio-dorsally and anterio-ventrally, bathed in midgut enzymes. Partially digested fluid is drawn through the mouth by a powerful pharyngeal pump until all but fragments of cuticle have been ingested, at which point the bolus is ejected.

Nodes and stems **G**-**N** map the evolution of the mandibles and labrum-epipharynx in the Cicindelitae.

Lineage **G** Evolution of the amblycheiline mandibles involves principally enlargement of the terebral tooth 1 (Figures 6C-D, **tt 1-1**), which becomes tricuspidate (**tt 1-1 – tt 1-3**). Within the Amblycheilini, the scrobal setae are retained in *Amblycheila*, but lost in the other genera (Figures 7A-B and 8A-B), and the slight down-curvature of the terebra is reduced (Figures 6C-D). The anterior margin of the labrum is variously modified, the median extension truncate (Figure 9A), or narrowly notched (Figure 9C), or broadly emarginate (Figure 9E).

Node **H** Evolution of the remaining lineages (**H-N**) of the Cicindelitae. The basic mandibular and labral-epipharyngeal features are those of the Amblycheilini, outlined above, except terebral tooth 1 consists only of a single large cusp.

Lineage **I** Evolution of the manticorine mandibles (Lineage **I**) involves marked ventral curvature of the terebra (Figures 10C-D). The mandibles of *Mantica* and female *Manticora* are otherwise very amblycheiline-like, but those of males are remarkably hypertrophied, with an especially elongate incisor tooth. Mandibular sexual dimorphism developed in numerous lineages throughout the phylogenetic history of tiger beetles, but became most pronounced in the lineage leading to *Manticora*, in which the males developed tremendously elongate mandibles, especially in the incisor region. Mandibular dimorphism likely developed in concert with prolonged copulation, and mate-guarding and the fitting of the male mandible to the female metathoracic copulatory sulcus (Freitag 1974). Oberprieler and Arndt (2000: 75-76) report that even the hypertrophied mandibles of these beetles function without any apparent awkwardness during tandem locomotion, and thus their allometric scaling appears appropriate for this purpose. The anterior margin of the labrum becomes shallowly emarginate, and develops four or six crenulations.

Lineage **J** Evolution of the Megacephalini and Collyridini + Cicindelini (Lineages **K- N**). Mandibles of this lineage develop a more or less extensive diastema between terebral tooth 1 and anterior margin of the retinaculum (Figures 11A-F – 13A-F, **od**), and retain the scrobal setae in a few genera (e.g. *Megacephala*, *Pogonostoma*). The epipharynx undergoes slight differentiation with the row of the anterior parapedial setae extended anteriorly (Figures 13D, F; 17F).

Lineage **K** Evolution of the mandibles of Megacephalini includes development of a supplementary retinacular tooth (Figures 11A-F, **srt**), and hence a longer retinaculum. Within this lineage, the terebral teeth differentiate in number (one to three) among taxa, and between sexes of the same taxon (cf. Pearson and Vogler 2001: 273, Figure B-12). The number of labral setae is reduced from 10-12 to four. The anterior margin differentiates from slightly projected medially to virtually truncate (Figure 13C). The epipharynx undergoes slight differentiation with the row of anterior parapedial setae extended anteriorly (Figure 13D, **aps**).

Lineage **L** The common ancestor of the tribes Collyridini and Cicindelini. Compared to those of its adelphotaxon (the Megacephalini), the mandibles (Figures 12A-F – 16A-F) become slender and labrum-epipharynx is extensively enlarged. The labral anterior margin becomes crenate, with the setae inserted apically rather than dorsally (Figures 13E and 17A, C). Since the arboreal members of the group evolved from ground-dwelling ancestors, tiger beetles serve as an example of the taxon pulse hypothesis (Erwin 1985) although the primarily terrestrial genus *Cicindela* appears to have evolved from arboreal ancestors (Vogler and Barraclough 1998).

Lineage **M** The Collyridini. The mandibular retinaculum becomes more complex, with one or more additional cusps (Figures 12B, D, F). Within the tribe, the mandibles become markedly varied. We illustrate only what must be a relatively basal lineage– the genus *Pogonostoma*, with only two terebral teeth on both left and right mandible, and only one additional retinacular cusp on only one (right) mandible. Scrobal setae are lost in all collyridine lineages examined with the exception of *Pogonostoma*. Terebral teeth are reduced in number in, or lost from, the genera *Ctenostoma* Klug, *Tricondyla* Latreille, and *Collyris* Fabricius.

Lineage **N** The Cicindelini. (Observations based principally on exemplar specimens representing the genus *Therates* Latreille, *Oxycheila* Dejean, and *Cicindela* Linnaeus, arranged in evolutionary order of appearance, according to the reconstructed phylogeny of Vogler and Barraclough). Also examined: *Oxygonia gloriola* Bates.

Within this tribe, and as in the Collyridini, the mandibles become markedly varied, as shown in Figures 14A-F, 15A-F and 16A-F. The scrobe, narrow in the more basal lineages, is lost from the more highly derived *Cicindela*. The ancestral number of terebral teeth was probably three as in *Cicindela* (Figures 16A-F), becoming four as in *Oxycheila* (Figures 15A-F, **tt 1 - tt 4**), and reducing to one, as in *Therates* (Figures 14A-F, **tt 2**). The supplementary retinacular tooth re-evolves in *Therates* (Figures 14A-F, **srt**). The ventral groove, normally quite long (Figures 15E-F, **vg**), becomes shortened in *Cicindela longilabris* (Figures 16E-F, **vg**), and in other species of this genus (Pearson and Vogler 2001: 198, Figure 105). The position of the labral setae, though preapical in the more basal cicindelines, shifts back to the dorsal surface in the more recently evolved genus *Cicindela*. Also, in this genus, the number of labral setae in some species is markedly increased, and in others decreased from the ancestral cicindelite 10-12, giving an overall range of 4 to 36. The labrum-epipharynx, quite long in the earlier-evolved cicindeline lineages (Figures 17A, C), becomes shortened in *Cicindela* (Figure 17E).

Vogler and Barraclough (1998) argue that rate of diversification (based on numbers of extant species) increased from the basal amblycheilines and manticorines to the more derived megacephalines and collyridines, and then to an even greater extent in the cicindelines. They attributed this pattern to the broad geographic ranges of the pantropical collyridines and the cosmopolitan megacephalines and cicindelines, and to the role of collyridines and cicindelines as large-eyed, diurnal, visual predators. In our work, a similar pattern was observed, with a narrow range of mandible and labrum-epipharynx structure among the basal taxa, and a broad range, including increased complexity of the preoral mill, among the more derived taxa. It is tempting to suggest that mouthpart evolution also played a part in the diversification of the higher cicindelites, but we are also curious whether the currently restricted geographic ranges and low diversity of the amblycheilines and manticorines might be better interpreted as relictual.

## Conclusions, and suggestions for further research

Building on increasingly sophisticated phylogenetic hypotheses for the Geadephaga, and the Cicindelitae (e.g. Maddison et al. 1999), as well as the functional morphology of Evans (1965) and Evans and Forsythe (1985), we are able here to propose a system of names and homologies for the structures of the tiger beetle mandibles and labrum-epipharynx, and to map hypothesized evolutionary changes in these structures on a generalized tree for the group. We propose relatively few multiple gains or losses of features, as follows: scrobal setae are lost in some but not all amblycheilines, megacephalines except *Megacephala*, and collyridines except *Pogonostoma*; the supplementary retinacular tooth evolves twice, in the megacephalines and the cicindeline genus *Therates*; terebral teeth are lost in many collyridines and the cicindeline genus *Therates*, terebral teeth increase in number in many cicindelines; and the labrum acquires the plesiotypic shortened condition in some cicindelines.

Further research would benefit from the examination of additional taxa, both within the Cicindelitae and among other relatively basal lineages of the Carabidae, in order to better address questions such as: is the Carabitae indeed the sister group of the Cicindelitae, and is *Platychile* more closely related to the amblycheilines or to the megacephalines? Incorporation of mouthpart features as character systems in phylogenetic analyses is recommended, as are further studies of the biomechanics of tiger beetle feeding and the use of mandibles during mating and mate-guarding, since the evolutionary changes we hypothesize are difficult to interpret without an appreciation of the functional consequences of changes in mouthpart configuration. In this light, further studies are also needed to adequately characterize sexual dimorphism, asymmetry, and chirality among tiger beetle mouthparts.

## Appendix

List of names of tiger beetle taxa examined with Wild M3 and M5 stereomicroscopes for mandibular and/or labral-epipharyngeal features.

incertae sedis

*Platychile pallida* (Fabricius, 1801) (2 males)

Tribe Manticorini

*Manticora mygaloides* Thomson, 1859 (2 males, 2 females)

*Manticora latipennis* Waterhouse, 1837 (1 male, 1 female)

*Manticora tuberculata* (DeGeer, 1778) (3 males, 2 females)

Tribe Megacephalini

*Megacephala regalis* Boheman, 1848 (1 male)

*Phaeoxantha tremolerasi* (W. Horn, 1909) (1 female)

*Phaeoxantha wimmeri* Mandel, 1958(1 male, 1 female)

*Tetracha annuligera* Lucas, 1857 (1 male, 1 female)

*Tetracha virginica* (Linnaeus, 1767)(1 male, 1 female)

Tribe Collyridini

*Ctenostoma metallicum* (Laporte de Castelnau, 1834) (1 male)

*Ctenostoma unifasciatum* Dejean, 1831 (1 male, 1 female)

*Ctenostoma ichneumoneum* Dejean, 1826 (1 female)

*Collyris dohrnii* Chaudoir. 1860(1 female)

*Tricondyla* species? (3 males)

Tribe Cicindelini

*Therates erinnys* Bates, 1874 (1 male, 1 female)

*Pseudoxycheila ceratoma* Chaudoir, 1865 (1 male)

*Cheiloxya binotata* Laporte de Castelnau, 1833 (1 male)

*Dromica junodi* Péringuey, 1892 (2 males, 1 female)

*Oxygonia gloriola* Bates, 1872 (= *Oxygonia simplicipennis* Horn) (1 male)
